# Overcoming erlotinib resistance in EGFR mutation-positive lung adenocarcinomas through repression of phosphoglycerate dehydrogenase: Erratum

**DOI:** 10.7150/thno.58558

**Published:** 2021-02-09

**Authors:** Jiang-Kai Dong, Hui-Min Lei, Qian Liang, Ya-Bin Tang, Ye Zhou, Yang Wang, Shengzhe Zhang, Wen-Bin Li, Yunguang Tong, Guanglei Zhuang, Liang Zhang, Hong-Zhuan Chen, Liang Zhu, Ying Shen

**Affiliations:** 1 Department of Pharmacology and Chemical Biology, Shanghai Jiao Tong University School of Medicine, Shanghai 200025, China; 2 Shanghai Universities Collaborative Innovation Center for Translational Medicine, Shanghai 200025, China; 3 State Key Laboratory of Oncogenes and Related Genes, Renji-Med X Clinical Stem Cell Research Center, Renji Hospital, Shanghai Jiao Tong University School of Medicine, Shanghai 200127, China; 4 Basic Medical College, Xinxiang Medical University, Xinxiang 453003, Henan, China; 5 Department of Medicine, Cedars-Sinai Medical Center, University of California Los Angeles School of Medicine, Los Angeles, CA 90048, United States

“HCC827ER9” was inadvertently mistyped as “HCC827ER4” in Figure 1D. The correction has now been made online. This corrigendum does not affect any results or conclusions of the paper. The authors sincerely apologize for any confusion and inconvenience may have caused.

## Figures and Tables

**Figure 1:**
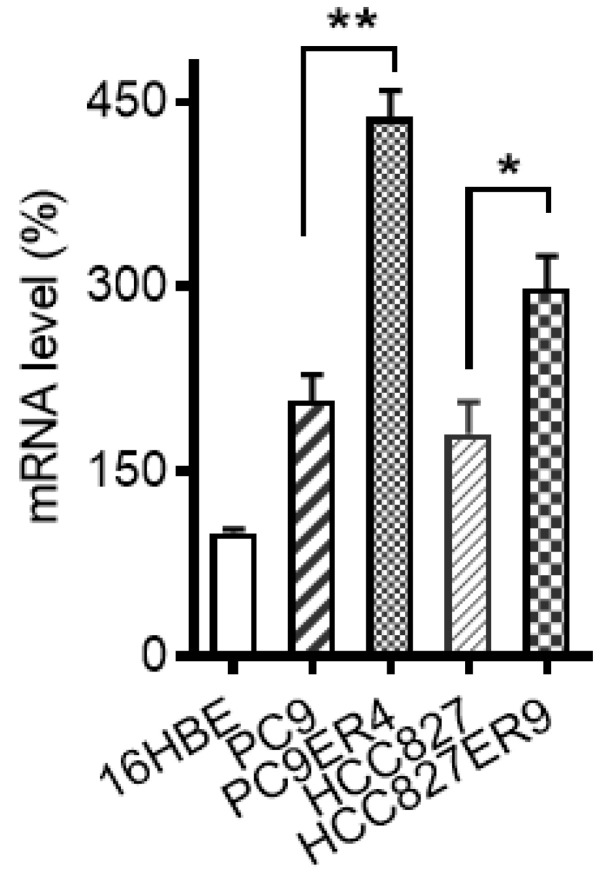
D. Corrected image

